# Intercellular Propagation and Aggregate Seeding of Mutant Ataxin-1

**DOI:** 10.1007/s12031-021-01944-1

**Published:** 2021-11-26

**Authors:** Haoyang Huang, Nicholas Toker, Eliza Burr, Jeff Okoro, Maia Moog, Casey Hearing, Sarita Lagalwar

**Affiliations:** Neuroscience Program, Skidmore College, Saratoga Springs, NY USA

**Keywords:** Ataxin-1, ATXN1, Spinocerebellar ataxia type 1, TNTs, Aggregation, Propagation

## Abstract

Intercellular propagation of aggregated protein inclusions along actin-based tunneling nanotubes (TNTs) has been reported as a means of pathogenic spread in Alzheimer’s, Parkinson’s, and Huntington’s diseases. Propagation of oligomeric-structured polyglutamine-expanded ataxin-1 (Atxn1[154Q]) has been reported in the cerebellum of a Spinocerebellar ataxia type 1 (SCA1) knock-in mouse to correlate with disease propagation. In this study, we investigated whether a physiologically relevant polyglutamine-expanded ATXN1 protein (ATXN1[82Q]) could propagate intercellularly. Using a cerebellar-derived live cell model, we observed ATXN1 aggregates form in the nucleus, subsequently form in the cytoplasm, and finally, propagate to neighboring cells along actin-based intercellular connections. Additionally, we observed the facilitation of aggregate-resistant proteins into aggregates given the presence of aggregation-prone proteins within cells. Taken together, our results support a pathogenic role of intercellular propagation of polyglutamine-expanded ATXN1 inclusions.

**Supplementary Information:**

The online version contains supplementary material available at 10.1007/s12031-021-01944-1.

## Introduction

Spinocerebellar ataxia type 1 (SCA1) is an autosomal dominant, progressive, and fatal neurodegenerative disease that primarily affects cerebellar Purkinje cells and brainstem nuclei, causing cerebellar syndromes including uncoordinated movement, gait disturbances, dysarthria, and dysphagia (Schut and Haymaker 1951; Matilla-Dueñas et al. 2008). As a polyglutamine (polyQ) disease, the main contributor to SCA1 is the expansion of CAG trinucleotide repeats coding for glutamine residues in the SCA1 gene product, ataxin-1 (ATXN1). Unaffected ATXN1 alleles contain 6 to 42 interrupted repeats, while affected alleles contain uninterrupted repeat tracts ranging from 39 to 82 residues (Orr et al. 1993; Chung et al. 1993; Zoghbi and Orr 2009; Kraus-Perrotta and Lagalwar 2016). The length of polyQ expansion directly correlates to disease severity and inversely correlates to age of onset.

The pathological hallmark of SCA1 is the formation of insoluble inclusions of mutant ATXN1 protein in extracerebellar neurons (Skinner et al. 1997; Klement et al. 1998; Watase et al. 2002). Previous work has characterized mutant ATXN1 nuclear inclusions as containing ubiquitin-conjugated substrates (Skinner et al. 1997) and the 20S proteasome subunit (Cummings et al. 1999). Cytoplasmic inclusions contain autophagic markers (Iwata et al. 2005), and are degraded upon autophagic activation (Berger et al. 2006). Taken together, these studies suggest that accumulation of aggregated ATXN1 disrupts autophagic and proteasomal degradation systems (Kohiyama and Lagalwar 2015).

However, as with other aggregation-prone neurodegenerative diseases, a clear causative role of inclusions in SCA1 toxicity remains inconclusive, particularly in light of the observation that unaffected neurons are more prone to inclusions while the most vulnerable neurons are spared (Klement et al. 1998). Notably, evidence suggests that soluble oligomers of mutant ATXN1 correlate with disease progression (Lasagna-Reeves et al. 2015a, b) and are capable of seeding and propagation in a mouse model of SCA1 (Lasagna-Reeves et al. 2015b).

In the present study, we characterize the structural and biochemical morphology of nuclear inclusions composed of a clinically relevant mutant ATXN1 construct (ATXN1[82Q]) in a human cerebellar-derived cell culture model of SCA1. Additionally, we observe the presence of inclusions in the cytoplasm following increased inclusion density, and eventual propagation into neighboring cells. Our results provide support for the dynamic propulsion of ATXN1 inclusions in SCA1.

## Material and Methods

### Cell Lines

Daoy human medulloblastoma cells (HTB-186) and Neuro2A mouse neuroblastoma cells (N2A, CCL-131) were purchased from American Type Culture Collection (ATCC; Manassas, MA). Stably transfected Daoy cells expressing *RFP-ATXN1[82Q; S776]-IRES-YFP*, *RFP-ATXN1[82Q; S776A]-IRES-YFP *or *RFP-ATXN1[30Q; S776]-IRES-YFP*) (Park et al. 2013) were a generous gift from the Orr lab at the University of Minnesota and the Zoghbi lab at Baylor College of Medicine. All cell lines were maintained in DMEM-high glucose (4.5 g/L) media supplemented with 10% fetal bovine serum, 1% penicillin/streptomycin, and 1% L-glutamine at 37ºC/5% CO_2_.

### Autophagy Assays

Daoy 30Q-RFP cells or Daoy 82Q-RFP cells were grown in culture and plated onto Nunc Lab Tek II 8-well chamber slides (ThermoFisher Scientific 154,534; Waltham, MA). The state of ATXN1 aggregation during the induction and inhibition of autophagy was determined using an autophagy assay kit (AbCam ab139484; Cambridge, MA). Briefly, 18 h following plating, cells were treated for 24 h with 500 nM rapamycin, dissolved in dimethylsulfoxide (DMSO; Sigma-Aldrich D2650;St. Louis, MO), and 60 µM chloroquine, dissolved in distilled deionized water, DMSO vehicle control or left untreated. Cells were stained for autophagy following the kit instructions and imaged on a FLoid Cell Imaging Station (ThermoFisher Scientific). Counts were performed from two randomly selected non-overlapping fields per chamber from each of three independent experiments.

### Cell Tracker Loading

Wild type Daoy cells were loaded with Molecular Probes CellTracker Green CMFDA dye (ThermoFisher Scientific C2925), and wild type N2A cells were loaded with QTracker 655 fluorescent Qdot nanocrystals (ThermoFisher Scientific Q25021MP) according to manufacturer’s instructions. Following fluorescence labeling and adherence, cells were harvested with Gibco TrypLE Express (ThermoFisher Scientific 12,605,036), and co-cultured with Daoy 82Q-RFP cells or wild type N2A cells that had been transiently transfected with eGFP-ATXN1[85Q], respectively. Cells were imaged on the FLoiD and Fluoview 1200 scanning confocal microscope (Olympus; Center Valley, PA).

### Plasmid Construction and Transfection

pEGFP-C2 plasmids inserted with *ATXN1[32Q]-S776, ATXN1[85Q]-S776* or *ATXN1[85Q]-A776* were provided as a generous gift by the Orr lab (University of Minnesota). Target cells were plated at a concentration of 20,000 cells/mL in Lab Tek II 8-well chamber slides and incubated overnight. Plasmid transfection was achieved with X-Treme Gene HP DNA Transfection Reagent (Sigma-Aldrich) according to the manufacturer’s instructions [0.8 μg DNA, 2 μg X-Treme gene transfection reagent, 4 h incubation in Opti-MEM (Gibco) media]. Transfection efficacy was determined after four hours using a FLoiD cell imaging station equipped with GFP and RFP filters.

### Immunocytochemistry

Harvested cells were plated in 8-well chamber slides (Lab Tek II) and allowed to grow for 3 days. Cells were fixed in ice cold methanol solution (10% MES Buffer (100 mM MES, pH 6.9, 1 mM EGTA, 1 mM MgCl_2_), 90% methanol) for 5 min on ice, washed with PBS, and blocked with 3% goat serum/0.5% Triton X-100/PBS for 15 min. Primary antibody (see antibody dilution table) was added for 3 h at 37 °C, and washed off. Secondary antibodies were added for either 1 h at 37 °C or overnight at 4 °C. DAPI nuclear stain was added via ProLong Gold antifade mounting medium (ThermoFisher P36931). Bright field images were acquired with the EVOS Cell Imaging System and the Olympus IX83 inverted microscope. Fluorescent images were taken from the EVOS Cell Imaging System equipped with RFP and GFP filters and the Olympus Fluoview 1200 Laser Scanning confocal microscope.

Antibodies dilution table (ICC and IHC).

**Table:** 

Antibody	Target	Species	Source	Dilution
11NQ	Total ATXN1, N-terminus	Rabbit	Orr lab, University of Minnesota	1:500 (ICC) 1:1000 (IHC)
11750	Total ATXN1, C-terminus	Rabbit	Orr lab, University of Minnesota	1:1000 (ICC)
PN1168	ATXN1[p-S776]	Rabbit	Orr lab, University of Minnesota	1:1000 (ICC)
F11G3	Oligomeric structure	Mouse	Sigma-Aldrich MABN1839	1:500 (ICC)
∝ -β-actin	β-actin	Mouse	AbCam ab8227	1:500 (ICC) 1:500 (IHC)
∝ -β-tubulin	β-tubulin	Mouse	Thermo Fisher 32–2600	1:1000 (ICC)
∝ -mouse- Alexa Flour-488	Mouse primary antibodies	Goat	Molecular Probes A28175	1:1000 (ICC) 1:500 (IHC)
∝ -rabbit- Alexa Flour-594	Rabbit primary antibodies	Donkey	ThermoFisher R37119	1:1000 (ICC) 1:500 (IHC)

## Results

To evaluate the potential seeding and propagation activity of ATXN1 aggregates in real time, we turned to a previously described cell culture model of SCA1 (Park et al. 2013). In this model, human cerebellar-derived medulloblastoma Daoy (ATCC HTB-186) cells were stably transfected with RFP-tagged ATXN1 constructs, containing a downstream IRES_YFP site allowing for independent translation of YFP at selectively low levels that were originally selected for flow cytometry studies (Park et al. 2013). YFP emission data was not collected in the current study; however, potential signal was screened for throughout the current study.

### ATXN1[82Q] Aggregate Formation in Daoy Cells

Aggregate promotion of mutant ATXN1 containing an expanded polyQ region was assessed by live cell imaging (Supplementary Fig. 1). RFP-ATXN1[30Q] protein is nuclear-localized and diffuse (Supplementary Fig. 1A), while the RFP-ATXN1[82Q] expanded polyQ region promotes aggregate formation. After 3 days in culture, cells with diffuse nuclear expression can be seen along with cells containing small, medium, and large size aggregates (Supplementary Fig. 1B). Previous research (Park et al. 2013; Carlson et al. 2009; Jorgensen et al. 2009; Jorgensen et al. 2007; Lai et al. 2011; Perez Ortiz et al. 2018) identified a second molecular attribute, phosphorylation of the ATXN1 Serine 776 site, that promotes aggregate formation specifically through 14–3-3 stabilization. Expression of phospho-resistant ATXN1[82Q-A776], in which the Serine 776 is mutated to an Alanine, is diffuse throughout the cytoplasm and nucleus (Supplementary Fig. 1C). Notably, expression levels of the ATXN1[82Q-A776] protein are low, as the unprotected protein is vulnerable to protease cleavage when 14–3-3 is absent.

Daoy cells double by mitosis every 33.6 h (Jacobsen et al. 1985); therefore, 4 days in culture in standard growth media results in approximately 6 times the number of cells. Increased cell density from day 1 (Supplementary Fig. 1D) to day 4 (Supplementary Fig. 1E) enhances aggregate formation among RFP-ATXN1[82Q] Daoy cells, resulting in fewer cells exhibiting diffuse expression.

**Fig. 1:**
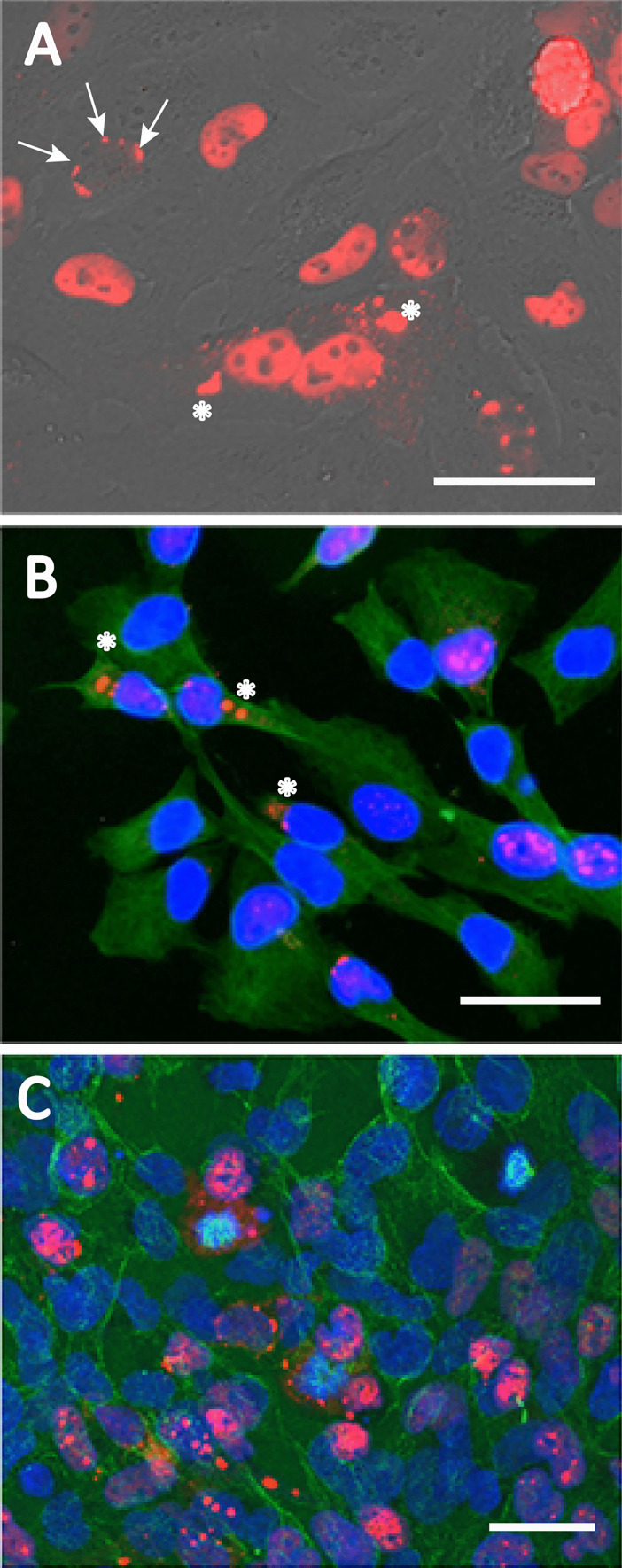
The presence of cytoplasmic RFP-ATXN1[82Q] aggregates in Daoy 82Q-RFP. **A** After 3 days in culture, RFP-ATXN1[82Q] aggregates begin to line up around the periphery of the nucleus (arrow) and transport out of the nucleus (asterisks). Extranuclear aggregates (asterisks) of total ATXN1 (**B**, 11750 antibody, red), or phospho-ATXN1 (**C**, PN1168 antibody, red) stained with tubulin (**B**–**C**, anti-β-tubulin, green), and DAPI (**B**–**C**). Size markers refer to 50 µm

In order to assess the structural identity of ATXN1[82Q] aggregates, we co-stained the aggregates with the 11750 antibody that recognizes total ATXN1 (red) and F11G3, which recognizes oligomeric structure (green). A large subset of ATXN1 co-localized with F11G3, while smaller subsets of ATXN1 + /F11G3 − and ATXN1 − /F11G3 + can be seen (Supplementary Fig. 1F). Next, we co-stained the aggregates with F11G3 (green) and the Serine 776-phosphorylation specific ATXN1 antibody PN1168 (red). Phosphorylated ATXN1 presents in both diffuse and aggregated forms, while the cells with aggregated phosphorylated ATXN1 show small amounts of diffuse proteins. Surprisingly, only a small subset of phosphorylated ATXN1 is oligomeric, and a large amount of oligomeric structures do not overlap with phosphorylated ATXN1 (Supplementary Fig. 1G).

### Manipulation of Autophagy and UPS Alters ATXN1 Aggregate Formation

To verify that normal cellular means for degrading and clearing misfolded proteins are disrupted by mutant ATXN1 expression, we manipulated the autophagic and ubiquitin-proteasomal systems (UPS) in our Daoy lines and noted the effects on ATXN1 aggregation. Live, untreated ATXN1[30Q] cells display autophagic activity, as determined with a marker of autophagy (Supplementary Fig. 2A, green). Treatment of ATXN1[30Q] cells with 500 nM rapamycin, an mTOR inhibitor, and inducer of autophagy (Berger et al. 2006; Noda and Ohsumi 1998; Blommaart et al. 1995) reduces the ATXN1[30Q] signal, indicating a reduction of wild-type ATXN1 protein (Supplementary Fig. 2B). Treatment of ATXN1[30Q] cells with 60 µM chloroquine, as a means of arresting the cells in late-stage autophagy, promotes large aggregate formation of the non-aggregation prone ATXN1[30Q] protein (Supplementary Fig. 2C).

Untreated ATXN1[82Q] aggregates do not typically co-localize with ubiquitin, and ubiquitin levels in ATXN1[82Q]-expressing cells is low (Supplementary Fig. 2D). However, inhibition of the proteasome with 100 nM lactacystin treatment enhances ubiquitin expression (green) and promotes ubiquitination of a subset of ATXN1[82Q] aggregates (asterisks) (Supplementary Fig. 2E). Unlike ATXN1[30Q], induction of autophagy with 500 nM rapamycin does not clear ATXN1[82Q] protein. Rather, it appears to enhance aggregate formation (Supplementary Fig. 2F). Arrest of late-stage autophagy in ATXN1[82Q] cells with 60 µM chloroquine further promotes large aggregate formation of the mutant protein (Supplementary Fig. 2G). Quantitative analysis of ATXN1[82Q]-expressing cells treated with rapamycin or chloroquine revealed that both treatments resulted in a re-distribution of diffuse nuclear ATXN1[82Q] protein into aggregated ATXN1[82Q] (Supplementary Fig. 2H; DMSO is a vehicle-control for rapamycin treatment; chloroquine was dissolved in water).

### ATXN1 Nuclear Aggregates Transport to the Cytoplasm

Previous work by Irwin et al. (Irwin et al. 2005) established a lack of nuclear export of aggregated eGFP-ATXN1[85Q] protein, compared to wild-type eGFP-ATXN1[26Q], in HeLa cells. However, in our Daoy models, nuclear export of aggregates, even large aggregates, is a common occurrence. It is quite possible that disassembly of the nucleus during mitosis may be the primary driver of this form of nuclear export. While we would expect to see a similar phenomenon in HeLa cells, recent work suggests that only a small fraction of HeLa cells within a colony may be immortal and involved in colony growth (Sato et al. 2016). However, we do note that inhibition of mitosis with 100–500 nM taxol arrested Daoy doubling, but did not prevent extranuclear expression of RFP-ATXN1[82Q] aggregates (data not shown), suggesting that there may be a non-mitotic mechanism allowing for nuclear export of aggregates.

Extranuclear RFP-ATXN1[82Q] aggregate presence is visible after 3 days in culture (Fig. 1). In live cells, nuclear aggregates form from diffuse RFP-ATXN1[82Q] prior to lining up along the inner perimeter of the nucleus (Fig. 1A, arrow). The lining up of aggregates was not visible in mitotic cells undergoing nuclear division. Next, aggregates can be seen in abundance outside of the nucleus (Fig. 1A, asterisks). Staining for tubulin (green), total ATXN1 (red), and DAPI confirms the presence of small and large cytoplasmic aggregates (asterisks) and intact nuclei (Fig. 1B). To confirm that phosphorylation of ATXN1 at its serine-776 site does not prevent cytoplasmic aggregate formation, we stained for ATXN1-(p)S776 (red), tubulin (green), and DAPI. Phosphorylated ATXN1[82Q] aggregates (asterisks) are found outside the nucleus in similar abundance (Fig. 1C).

### ATXN1 Aggregates Can Enter Acceptor Cells

**Fig. 2:**
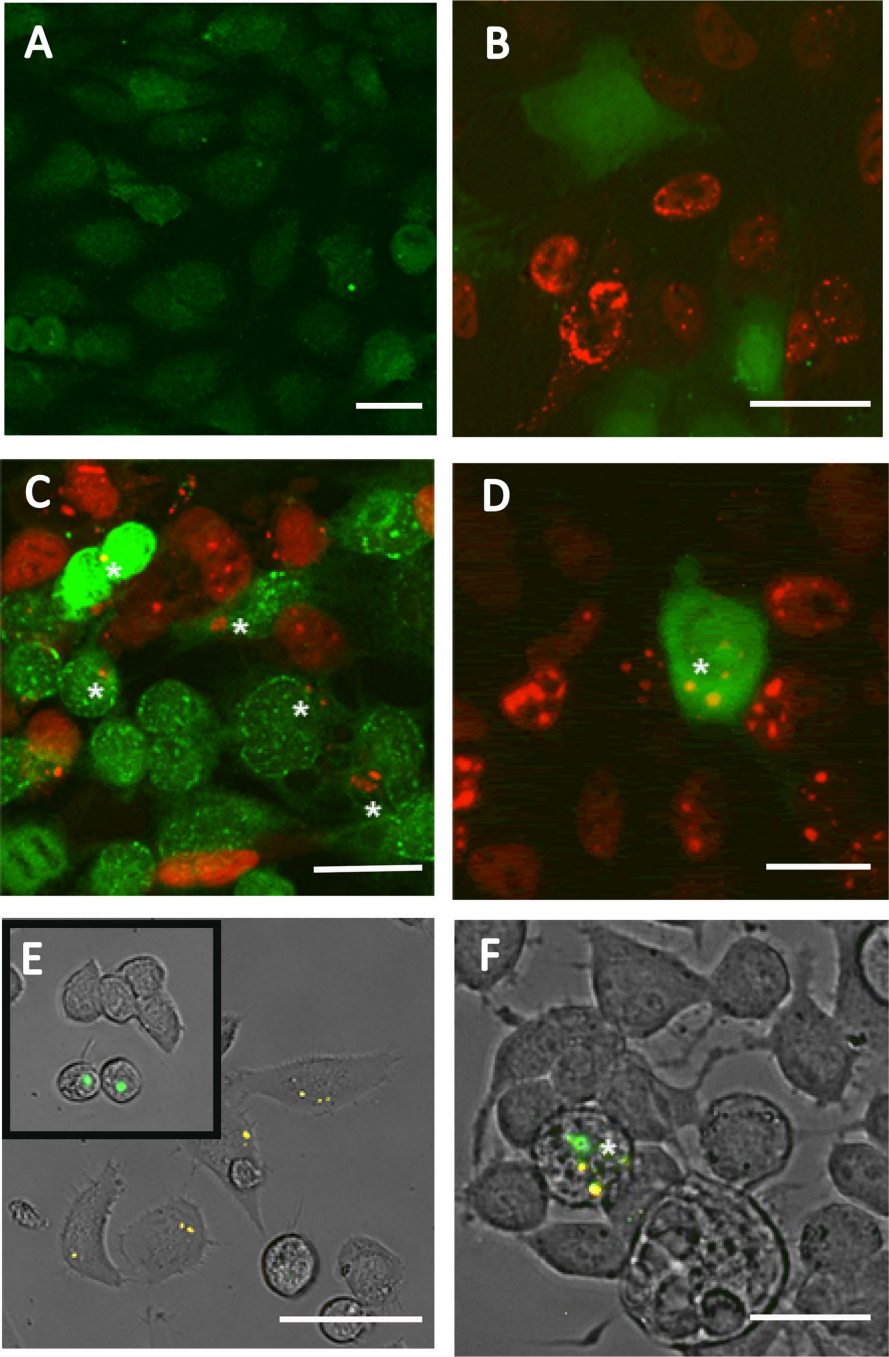
ATXN1 aggregates can enter into acceptor cells in co-cultures. **A** Cell Tracker™ Green CMDFA dye loaded into wild type Daoy cells prior to co-culturing fills the cytoplasm and nucleus in a diffuse, lightly punctate manner. **B** One day following co-culturing Cell Tracker-loaded Daoy cells with Daoy RFP-ATXN1[82Q] cells, the two cultures remain distinct. **C** Three days following co-culturing, RFP-ATXN1[82Q] aggregates are seen in Cell Tracker-loaded cells (asterisks). **D** Zoomed-in image of a Cell Tracker-loaded cell (asterisk) containing 4 RFP-ATXN1[82Q] aggregates. **E** N2A cells were loaded with Qtracker™ 655 (yellow), which appear as small cytoplasmic nanoparticles. Qtracker-loaded N2A cells were co-cultured with N2A cells transiently transfected with eGFP-ATXN1[85Q] (inset). **F** Three days following co-culturing, eGFP-ATXN1[85Q] aggregates can be seen in Qtracker-loaded cells (asterisk). Size markers refer to 50 µm

The presence of extranuclear aggregates following nuclear localization of aggregates led to the question of whether cytoplasmic aggregates could transfer from one cytoplasm to another. To address this question, we prepared “acceptor” cells by loading non-transfected Daoy cells with Cell Tracker™ Green CMDFA dye (Fig. 2A). Once loaded, the dye does not leak out of cells. The loaded acceptor cells were then co-cultured with RFP-ATXN1[82Q]-expressing Daoy cells, the “donor” cells (Fig. 2B). On day three of co-culturing, RFP-ATXN1[82Q] aggregates are noticeable in 5% of all Cell Tracker loaded “acceptor” cells (Fig. 2C–D).

To assess whether this phenomenon is partially or fully due to the cell type, cell species, fluorophore, donor dye, or the stable nature of transfection, we repeated this experiment in mouse Neuro2a neuroblastoma cells ((ATCC CCL-131); N2a). eGFP-ATXN1[85Q] was transiently transfected into N2a cells and cultured (Fig. 2E, inset). A separate batch of non-transfected N2a cells were loaded with QTracker™ 655 nanoparticles. As with the Cell Tracker dye, QTracker does not leak out of loaded cells. The two batches of cells were co-cultured; 3 days following co-culture, eGFP-ATXN1[85Q] aggregates (green) appear in QTracker (yellow) loaded cells (Fig. 2F).

### ATXN1 Aggregates Propagate Between Cells Along Actin-Based Connections

**Fig. 3:**
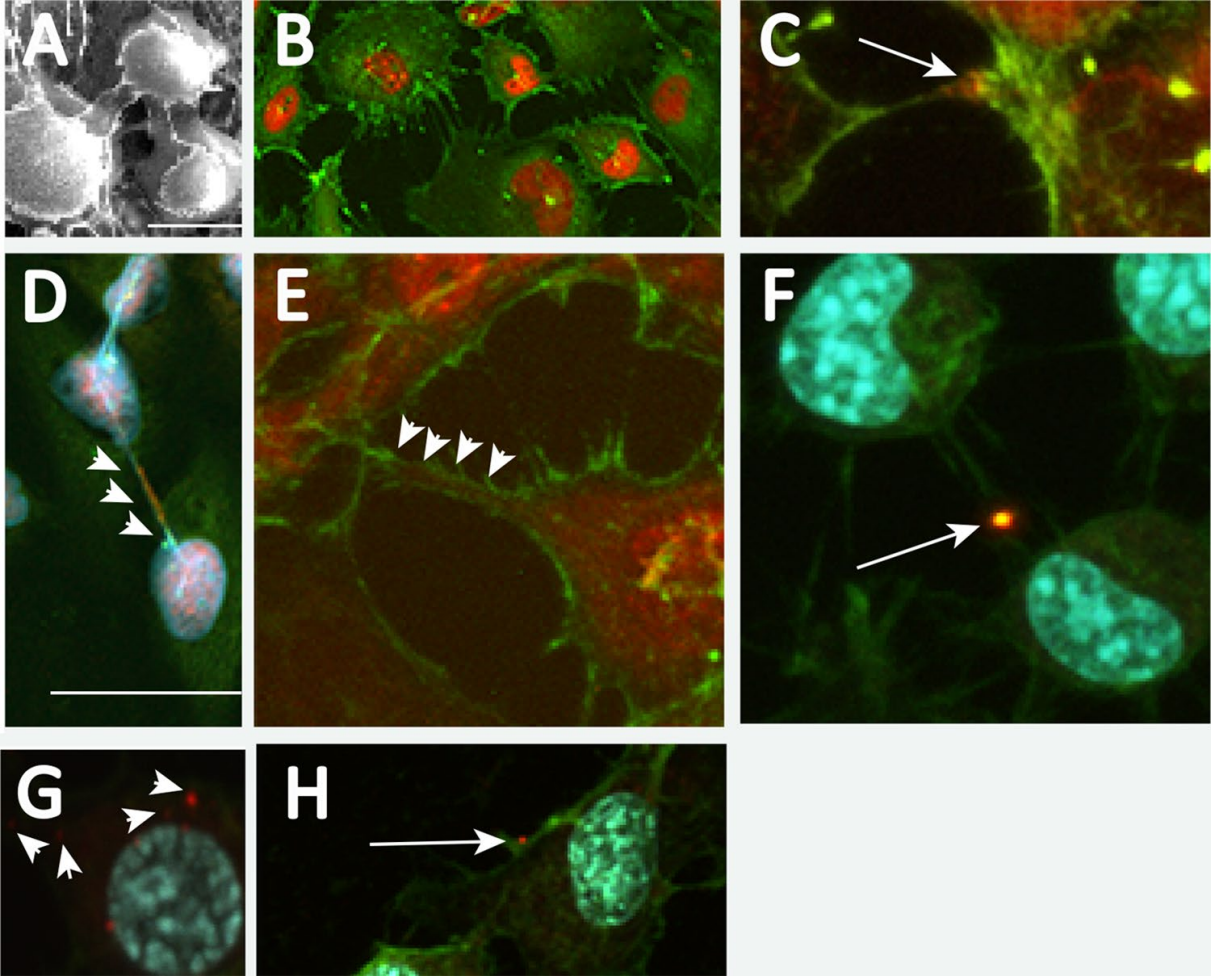
ATXN1 aggregates leave cells and propagate along actin-based intercellular connections. **A** Scanning electron microscopy image of intercellular connections among Daoy cells in culture. Size marker is 10 µm. **B** Actin-labeling (green) of Daoy RFP-ATXN1[82Q] cells depicts actin-based intercellular connections. **C** After 3 days in culture, a large RFP-ATXN1[82Q] aggregate (arrow) can be found along an actin-based intercellular connection. **D** Multiple small RFP-ATXN1[82Q] aggregates (arrowheads) along an actin-based intercellular connection (anti-actin, green; DAPI. Size marker is 50 µm). **E** Actin-positive (green), tube-like connection between Daoy cells is filled with RFP-ATXN1[82Q] proteins (arrowheads). **F** Large ATXN1[85Q] (11NQ antibody, red) aggregate (arrow) on an actin-positive (green) intercellular connection between N2A cells. **G** Multiple small ATXN1[85Q] (11NQ antibody, red) aggregates (arrowheads) accumulate near the actin cortex (green; DAPI, white) of a N2A cell. **H** Small ATXN1[85Q] (11NQ antibody, red) aggregate (arrow) on extracellular actin (green)

Actin-based tunneling nanotubes of varying lengths have been identified as cell-to-cell cytoplasmic-filled passages for transfer of aggregation-prone proteins including polyglutamine-expanded huntingtin protein (Costanzo et al. 2013; Zhu et al. 2015; Abounit et al. 2016a, b; Dieriks et al. 2017; Chastagner 2020; Dilsizoglu Senol et al. 2019; Victoria and Zurzolo 2017; Rostami et al. 2017; Vilette et al. 2018). In culture, Daoy cells form a thin, uniform monolayer with cell-to-cell connections (Fig. 3A). Immunocytochemistry reveals that actin labels these cell-to-cell connections (Fig. 3B). To determine if actin-labeled TNTs provided a mode of passage for expanded ATXN1 protein aggregates, RFP-ATXN1[82Q]-expressing Daoy cells were grown in culture for 3 days, and then labeled for actin (green). Individual aggregates (Fig. 3C) and streams of RFP-ATXN1[82Q] protein (Fig. 3D, E) were found along the TNTs. Individual aggregates of ATXN1[85Q] (red) transiently transfected into cultured N2a cells were also found along actin-labeled TNTs (green) following 3 days of culture (Fig. 3F–H).

#### ATXN1 Aggregates Incorporate Non-aggregate Prone ATXN1 Into Aggregates

**Fig. 4:**
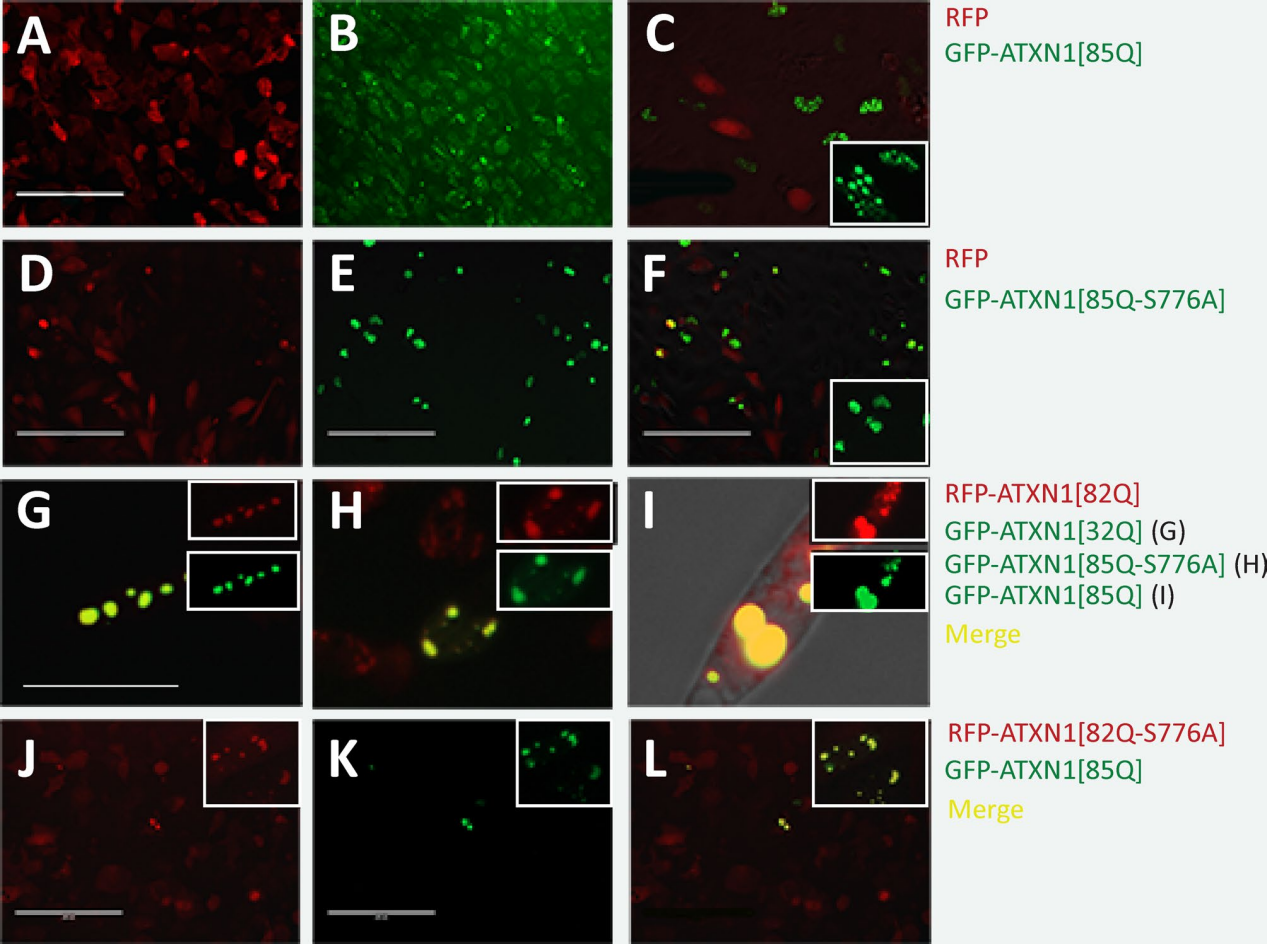
ATXN1 aggregates can seed non-aggregate prone ATXN1 into aggregate formation. **A**–**C** Aggregation prone GFP-ATXN1[85Q] transiently transfected into control Daoy cells stably expressing RFP shows little overlap between green ATXN1 aggregates and RFP protein. RFP shows little aggregation. Size marker is 200 µm. **C** Zoomed-in merged image of **A** and **B**; inset shows close-up of GFP-ATXN1[85Q] aggregates. **D**–**F** Aggregation-resistant GFP-ATXN1[85A-S776A] shows slight aggregation when over-expressed in Daoy-RFP cells through transient transfection. RFP does not aggregate. **F** Merged image of **D** and **E**; inset shows close-up of GFP-ATXN1[85Q-S776A] protein. Size marker is 200 µm. Hybrid aggregates of RFP-ATXN1[82Q] with GFP-32Q (**G**), GFP-ATXN1[85Q-A776] (**H**), and GFP-ATXN1[85Q] (**I**). Insets show single channel images. Insets show close-up image of aggregates. Size marker is 50 µm. Transmitted light image shown in **I**. **J**–**L** Stably transfected RFP-ATXN1[82Q-A776] is aggregation-resistant but aggregates in the presence of transiently transfected RFP-ATXN1[85Q] aggregates. Insets show close-up aggregates from other images. Size marker is 200 µm

To determine if aggregation-prone ATXN1 can seed or incorporate non-aggregation prone ATXN1 into aggregates, we transiently transfected GFP-ATXN1 constructs into RFP-ATXN1 stably transfected Daoy cells. As a control, free RFP-expressing Daoy cells were transfected with GFP-ATXN1[85Q] (Fig. 4A–C). GFP-ATXN1[85Q] readily forms aggregates (Fig. 4B), while free RFP remains diffuse (Fig. 4A, C). RFP does not co-localize with GFP-ATXN1[85Q] (Fig. 4C). As an additional control, RFP cells were transfected with non-aggregation prone GFP-ATXN1[85Q-S776A] (Fig. 4D–F). While some co-localization can be seen in yellow, expressions of both RFP and GFP-ATXN1[85Q-S776A] remain mainly diffuse (Fig. 4F).

In contrast, RFP-ATXN1[82Q]-expressing Daoy cells (Fig. 4G–I) transfected with aggregation-resistant GFP-ATXN1[32Q] (Fig. 4G), aggregation-resistant GFP-ATXN1[85Q-S776A] (Fig. 4H), or aggregation prone GFP-ATXN1[85Q] (Fig. 4I) result in incorporation of the GFP-labeled protein into the RFP-ATXN1[85Q] aggregate (insets reflect single channel images). Transfection of aggregation-resistant RFP-ATXN1[82Q-S776A]-expressing Daoy (Fig. 4J) with aggregation-prone GFP-ATXN1[85Q] (Fig. 4K) likewise results in incorporation of the aggregation-resistant protein into the GFP-labeled aggregate (inset shows additional instance of co-localization).

## Discussion

In the current study, we use a human cerebellar-derived medulloblastoma cell line to characterize three steps of the intercellular propagation of ATXN1 aggregates in Daoy cells: (1) the presence of cytoplasmic ATXN1 aggregates following nuclear aggregate formation, (2) extracellular propagation of ATXN1 aggregates via actin-based tunneling nanotubes, and (3) incorporation of aggregation-prone and aggregation-resistant ATXN1 proteins into seeded aggregate formations.

RFP-ATXN1[82Q] nuclear aggregates were readily observed in Daoy cells, while RFP-ATXN1[30Q] remained diffuse (Supplementary Fig. 1A–C), thus supporting established previous findings that an uninterrupted elongated polyQ region promotes the formation of aggregates (10.1038/40153; https://doi.org/10.1016/s0092-8674(00)81781-x). Immunocytochemical analysis of aggregates showed a colocalization of total ATXN1 protein and oligomers in RFP-ATXN1[82Q] Daoy cells, suggesting that a portion of ATXN1 protein adopted oligomeric structure in pathogenic cells (Supplementary Fig. 1F). We also saw colocalization of largely extranuclear, aggregated Ser776-phosphorylated ATXN1, and oligomeric protein in pathogenic cells (Supplementary Fig. 1G). Considering that Ser776 phosphorylation can stabilize the structure of ATXN1, the current results suggest that initial aggregation occurs among phosphorylated ATXN1 proteins. Furthermore, the presence of a subset of aggregates that are oligomeric may indicate motility, specifically by transport into neighboring cells. To this end, ATXN1 aggregates might not directly contribute to cellular toxicity but rather to the progression and propagation of the disease.

Unexpectedly, both cytoplasmic and nuclear ATXN1[82Q] aggregates were visible upon 3 days in culture (Fig. 1). The presence of cytoplasmic aggregates contrasted with previous work. In particular, Irwin et al. demonstrated in a FRAP live cell shuttle assay in HeLa cell bikaryon that GFP-ATXN1[84Q] — once transported into the nucleus — was incapable of nuclear export (Irwin et al. 2005). The same study found that GFP-ATXN1[26Q] could readily transport into and out of the nucleus following photobleaching (Irwin et al. 2005). Differences in our findings may be due to the difference in cell lines. Daoy provides a human cerebellar-derived model that expresses endogenous human ATXN1 (Park et al. 2013), while HeLa contributes a foreign cellular environment for ATXN1 dynamics. Signaling pathways known to stabilize and degrade ATXN1 in mammalian Purkinje cells are conserved in the Daoy line (collectively shown in these studies: (Park et al. 2013; Jorgensen et al. 2007, 2009; Lai et al. 2011; Perez Ortiz et al. 2018; Kaytor et al. 2005; Lagalwar and Orr 2013)), indicating that Daoy cells are a viable means of studying ATXN1 properties in an intact cellular system. Additionally, GFP-ATXN1[84Q] was transiently transfected into HeLa cells (Irwin et al. 2005) while RFP-ATXN1[82Q] was stably transfected into Daoy cells. The constitutive and continuous nature of stable transfection may enhance nuclear formation of ATXN1 aggregates, thereby leading to higher concentrations of exported ATXN1 aggregates into the cytoplasm. Alternatively, we cannot exclude the possibility that given the proper conditions, ATXN1 aggregates might form spontaneously in the cytoplasm. The existence of the phospho-motif binding protein 14–3-3 in the cytoplasm provides a possible mechanism for cytoplasmic aggregation. 14–3–3 readily binds ATXN1 phosphorylated at the Ser776 site, blocking phosphatases that dephosphorylate ATXN1 and lead to rapid proteolytic degradation (Lai et al. 2011; Fu et al. 2000; Athwal et al. 2000). Possibly, the stabilization of mutant ATXN1 in the cytoplasm by 14–3–3 protein facilitates the formation of cytoplasmic inclusions. Supportive of this possibility is our finding that manipulation of cytoplasmic autophagy by rapamycin and chloroquine alters the distribution of nuclear diffuse and aggregated ATXN1[82Q] (Supplementary Fig. 2). While it can’t be ruled out that nuclear inclusions are processed through autophagy via degradation of the nuclear lamina (Dou et al. 2016), our present findings are congruous with previous work that identified evidence of autophagic flux in the cytoplasmic vacuoles of Purkinje cells from SCA1 ATXN1[82Q] transgenic mice (Kohiyama and Lagalwar 2015; Vig et al. 2009).

Furthermore, our co-culture study indicates that the ATXN1 aggregates appear in non-transfected “acceptor” cells co-cultured with either stably (Fig. 2A–D) or transiently (Fig. 2E, F) transfected “donor” cells. Therefore, ATXN1 aggregates, after being transported into the cytoplasm, can propagate to other cells (Jucker and Walker 2018; Jaunmuktane and Brandner 2020). In the current study, we investigate whether tunneling nanotubes (TNTs), an actin-rich intercellular networked structure for short-range transport of molecules (Rustom 2009; Gerdes et al. 2007; Gurke et al. 2008; Gerdes and Carvalho 2008; Baluska et al. 2004), are the primary pathway for such propagation. Intercellular actin-composed structures are extensively formed in Daoy and N2a cell culture (Fig. 3A–F), and the presence of small ATXN1 aggregates in such structures indicates that ATXN1 aggregates are capable of being transported via these structures (Fig. 3C–H). These results strongly suggest that small ATXN1 aggregates, possibly at the early pathological stage, can escape or be actively transported from the host via TNT-like actin transport.

Such observations resonate well with our transfection experiments, which show that aggregation-prone ATXN1 proteins recruited (1) non-aggregating ATXN1[32Q] proteins (Fig. 4G), (2) aggregation-resistant ATXN1[85Q-S776A] (Fig. 4H) and ATXN1[82Q-S776A] (Fig. 4J–L) proteins, and (3) aggregation-prone ATXN1[85Q] proteins into aggregates (Fig. 4I). Both non-aggregating or aggregation-resistant ATXN1 proteins are capable of forming aggregates in the presence of pre-existing aggregation-prone ATXN1 proteins, exhibiting prion-like activity. It is possible that aggregated ATXN1 proteins recruit surrounding proteins indiscriminately; however, the observation that GFP-labeled ATXN1 proteins are highly colocalized with RFP-ATXN1[82Q] aggregates (Fig. 4G–I) suggests that ATXN1 aggregates might exclusively recruit ATXN1 proteins.

Taken together, the current study utilized Daoy cells, a human cerebellar-derived model that expresses endogenous human ATXN1 to characterize in vitro behaviors of ATXN1 aggregates and reveals three important aspects of ATXN1 mediated SCA1 pathology: (1) the presence of ATXN1 aggregates in the cytoplasm after extended culture, (2) the cell-to-cell propagation of ATXN1 aggregates via TNT-like structures, and (3) prion-like seeding behaviors after entering non-transfected cells. These observations support a prion-like propagation mechanism for ATXN1 aggregates. This mechanism has been extensively investigated in other neurodegenerative diseases with aggregate-prone proteins including tau in Alzheimer’s disease (Abounit et al. 2016b; Chastagner 2020), huntingtin in Huntington’s disease (Costanzo et al. 2013), and alpha synuclein in Parkinson’s disease (Abounit et al. 2016a; Dieriks et al. 2017; Rostami et al. 2017). Considering that propagation is prevalent across different neurodegenerative diseases, it is likely mediated via similar pathways. In that case, the clear pathogenesis of ATXN1-mediated SCA1 may provide insight into the more complex mechanisms of Alzheimer’s and Parkinson’s disease.

## Supplementary Information

Below is the link to the electronic supplementary material.

Supplementary file1 (DOCX 339 KB)

## Data Availability

The authors will upload the data to a repository such as Figshare, or as recommended by the journal.
