# Supplementary material for: Intercellular Propagation and Aggregate Seeding of Mutant Ataxin-1
Source: J Mol Neurosci. 2021 Nov 26;72(4):708–18. doi: 10.1007/s12031-021-01944-1 (PMC8986690; doi:10.1007/s12031-021-01944-1)

**Supplementary Materials**

**Supplementary Figure 1.** Characterization of RFP-ATXN1[82Q] aggregates stably expressed in Daoy cells. A) RFP-ATXN1[30Q] stably expressed in Daoy cells is diffuse and localized to the nucleus. B) RFP-ATXN1[82Q] stably expressed in Daoy cells is primarily localized to the nucleus and can be diffuse (D), or aggregate into small (S), medium (M), or large (L) species. C) RFP-ATXN1[82Q-A776], in which the NLS serine is mutated to a phosphorylation-resistant alanine, is diffuse, expressed in the cytoplasm and nucleus, and unstable. D-E) Cell density increases aggregate expression as seen in cells plated on day 1 (D) compared to day 4 (E). F) Co-localization of total ATXN1[82Q] (11750 antibody, red) and oligomers (F11G3 antibody, green) in Daoy cells. G) Co-localization of ATXN1[82Q] phosphorylated at the Serine 776 residue (PN1168 antibody, red) and oligomers (F11G3 antibody, green) in Daoy cells. Size markers refer to 50 μm.


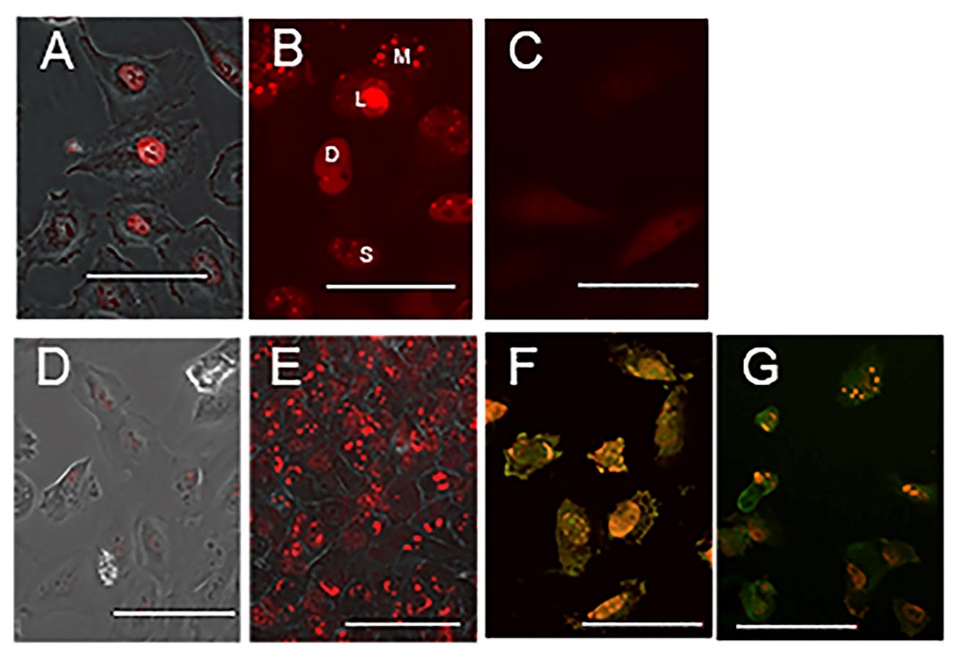


**Supplementary Figure 2.** Manipulation of autophagy and UPS alters ATXN1 aggregate formation. A) RFP-ATXN1[30Q] nuclear expression in Daoy cells shown with an autophagy marker (green). B) Induction of autophagy with 500 nM rapamycin treatment reduces RFP-ATXN1[30Q] expression. C) Arrest of late-stage autophagy by 60 µM chloroquine induces formation of RFP-ATXN1[30Q] into large aggregates. D) RFP-ATXN1[82Q] aggregates do not co-localize with ubiquitin aggregates (anti-ubiquitin, green) when treated with DMSO-vehicle. E) Proteasomal inhibition by 100 nM lactacystin treatment induces ubiquitination (green) of a subset (asterisks) of ATXN1[82Q] aggregates (11750 antibody, red). F-G) 24-hour treatment of Daoy cells one day after plating with 500 nM rapamycin (F) or 60 µM chloroquine (G) alters aggregation of RFP-ATXN1[82Q]. Autophagic marker is shown in green, DAPI is shown in blue. H) Induction of autophagy by 500 nM rapamycin or arrest of autophagy by 60 µM chloroquine redistributes diffuse, nuclear RFP-ATXN1[82Q] into aggregates. Error bars reflect SEM. ANOVA with Tukey’s multiple comparisons test. ** P < 0.01, *** P < 0.05. Size markers refer to 50 μm.


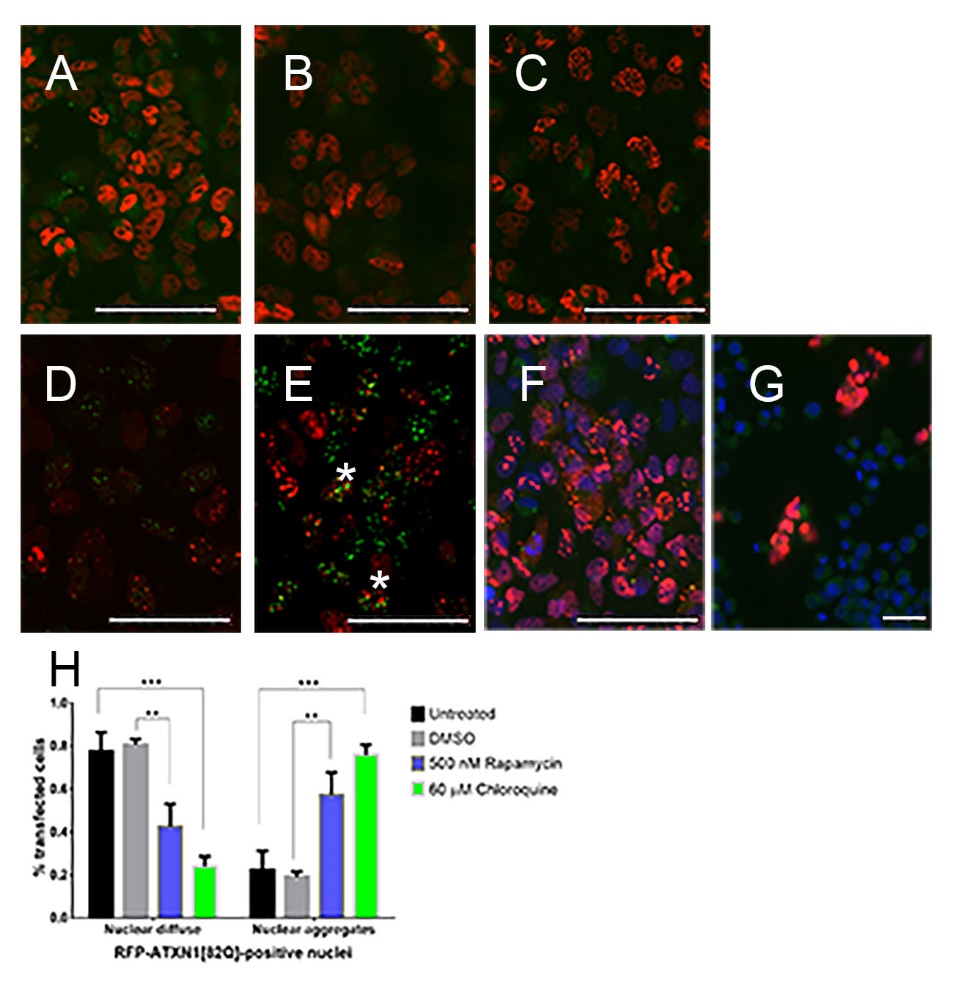

Supplement: Supplementary file 1 — Supplementary file1 (DOCX 339 KB) [file 12031_2021_1944_MOESM1_ESM.docx]
